# Resting-State Functional MRI Metrics in Patients With Chronic Mild Traumatic Brain Injury and Their Association With Clinical Cognitive Performance

**DOI:** 10.3389/fnhum.2021.768485

**Published:** 2021-12-27

**Authors:** Faezeh Vedaei, Andrew B. Newberg, Mahdi Alizadeh, Jennifer Muller, Shiva Shahrampour, Devon Middleton, George Zabrecky, Nancy Wintering, Anthony J. Bazzan, Daniel A. Monti, Feroze B. Mohamed

**Affiliations:** ^1^Department of Radiology, Jefferson Integrated Magnetic Resonance Imaging Center, Thomas Jefferson University, Philadelphia, PA, United States; ^2^Department of Integrative Medicine and Nutritional Sciences, Marcus Institute of Integrative Health, Thomas Jefferson University, Philadelphia, PA, United States

**Keywords:** traumatic brain injury, resting-state functional magnetic resonance imaging, fractional amplitude of low-frequency fluctuation, regional homogeneity, functional connectivity, cognitive performance

## Abstract

Mild traumatic brain injury (mTBI) accounts for more than 80% of people experiencing brain injuries. Symptoms of mTBI include short-term and long-term adverse clinical outcomes. In this study, resting-state functional magnetic resonance imaging (rs-fMRI) was conducted to measure voxel-based indices including fractional amplitude of low-frequency fluctuation (fALFF), regional homogeneity (ReHo), and functional connectivity (FC) in patients suffering from chronic mTBI; 64 patients with chronic mTBI at least 3 months post injury and 40 healthy controls underwent rs-fMRI scanning. Partial correlation analysis controlling for age and gender was performed within mTBI cohort to explore the association between rs-fMRI metrics and neuropsychological scores. Compared with controls, chronic mTBI patients showed increased fALFF in the left middle occipital cortex (MOC), right middle temporal cortex (MTC), and right angular gyrus (AG), and increased ReHo in the left MOC and left posterior cingulate cortex (PCC). Enhanced FC was observed from left MOC to right precuneus; from right MTC to right superior temporal cortex (STC), right supramarginal, and left inferior parietal cortex (IPC); and from the seed located at right AG to left precuneus, left superior medial frontal cortex (SMFC), left MTC, left superior temporal cortex (STC), and left MOC. Furthermore, the correlation analysis revealed a significant correlation between neuropsychological scores and fALFF, ReHo, and seed-based FC measured from the regions with significant group differences. Our results demonstrated that alterations of low-frequency oscillations in chronic mTBI could be representative of disruption in emotional circuits, cognitive performance, and recovery in this cohort.

## Introduction

Traumatic brain injury (TBI) is one of the most common neurological disorders, accounting for more than 10,000,000 annual deaths and hospitalizations worldwide (Roozenbeek et al., 2013; Sinke et al., 2021). It is characterized by cognitive and emotional deficits within the first few weeks after injury and may persist up to 3–6 months post injury, and in some cases for several years (Mayer et al., 2014, 2015; Komura et al., 2019). It has been reported that chronic symptoms from mild TBI (referred to as chronic mTBI although it is sometimes called post-concussion syndrome) may develop in some individuals. Such patients present with chronic cognitive, neurological, and behavioral symptoms including memory loss, depression, and other deficits in social functioning and reduced quality of life (Hiploylee et al., 2017). Resting-state functional magnetic resonance imaging (rs-fMRI) has been widely used in research to explore the intrinsic functional architecture between brain networks as the biomarkers of neurological and cognitive disorders (Jeter et al., 2013; Sheth et al., 2021).

Several approaches have been conducted using rs-fMRI to estimate brain functional connectivity (FC) and resting-state brain networks in TBI patients. These include conventional methods of seed-based connectivity analysis (Lemme et al., 2021), independent component analysis (Bittencourt-Villalpando et al., 2021), and graph theory (Van Der Horn et al., 2017). Spontaneous low-frequency oscillations (LFOs) is another technique that has been recently used to reveal brain spatiotemporal structure. LFOs of the rs-fMRI signals reflect spontaneous brain neural activity within a range of approximately 0.01–0.1 Hz as this frequency has been shown to be linked with neural fluctuations. Additionally, LFO amplitude has demonstrated encouraging test–retest reliabilities, particularly for the measures derived from gray matter compared with those derived from white matter, since this is less affected by physiological phenomena in gray matter (Zuo et al., 2010). Measures including fractional amplitude of low-frequency fluctuations (fALFF) and regional homogeneity (ReHo) have been shown to provide robust and reliable biomarkers for depicting regional properties of rs-fMRI data. The fALFF is the fast Fourier transform (FFT)-based indices of LFO amplitude. This is defined as a relative contribution of the power of low-frequency blood-oxygen-level-dependent (BOLD) signal fluctuations and assumed to reflect the magnitude of neural activity (Zuo et al., 2010; Golestani et al., 2017).

Likewise, the ReHo is calculated within the low-frequency fluctuations using Kendall’s coefficient of concordance (KCC). ReHo measures the similarity between the time series of a given voxel and that of its nearest neighbors in a voxel−wise manner. This method is appropriate to detect the local synchronization of LFOs, which presumably reflects local functional connectivity (Zang et al., 2004; Zou et al., 2008; Zuo and Xing, 2014; Zhao et al., 2018; Yang et al., 2020). An increasing number of studies have reported alteration of LFO amplitudes in a wide range of neurological and cognitive disorders including Alzheimer’s disease (Yang et al., 2018), Parkinson’s disease (Wang et al., 2020; Yue et al., 2020), brain aging (Jiao et al., 2019), neuropsychological disorders such as major depression and anxiety (Lin et al., 2020; Mo et al., 2020), and TBI (Palacios et al., 2013; Zhou et al., 2014).

Amplitude of low-frequency fluctuations (ALFF) and ReHo have been employed to detect spontaneous neural activity and local synchronization of neural activity corresponding to neurocognitive function in TBI patients. Recently, one study investigated ALFF among acute mild TBI (mTBI) patients compared with healthy volunteers and found higher ALFF in the patient group in the occipital and fusiform/lingual gyrus and decreased ALFF in the frontal lobe. However, inconsistent findings surrounding resting-state metrics have been reported in the literature; these could be attributed to several reasons, such as the stage and severity of the disease, the time of the study after injury, sample size, and heterogenous population phenotypes. For instance, a previous study compared fALFF between mTBI patients and healthy control (CN) and found decreased fALFF in the frontal, temporal, and occipital lobes in patients compared with CN. In addition, they applied seed-based FC analysis with a seed placed in the thalamic area and reported higher FC with the frontal, parietal, and occipital regions and decreased FC with the temporal lobe (Zhou et al., 2014).

A prior study conducted to examine ReHo in patients with mTBI found increased ReHo in the superior frontal and middle occipital regions and decreased ReHo in the inferior frontal, medial frontal, superior temporal, parahippocampus, supramarginal, and supplement motor cortex areas in the patients compared with CN. Moreover, they reported a significant correlation between cognitive function and ReHo in the superior frontal gyrus (Liu et al., 2018). ReHo reduction in the precentral/postcentral, supramarginal gyrus, and insula has also been measured and was positively correlated with cognitive ability in the left insula (Zhan et al., 2015).

Despite these various findings, rs-fMRI metrics have provided insight into the possible neurophysiological mechanism underlying brain injury (Meier et al., 2020). However, the current study is the first to investigate the alterations of LFO amplitude measurements in mTBI patients in the chronic stage (at least 6 months post injury) and to explore the relationship with neurocognitive ability. To that end, in this study we examined the alteration of fALFF and ReHo in chronic mTBI patients followed by secondary seed-based FC analysis using the regions that showed significant alteration in fALFF and ReHo. Lastly, we evaluated the possible correlation between neuropsychological assessments and rs-fMRI metrics within the patient group. We tested the hypothesis that brain injury would result in alteration of rs-fMRI measurements and in association with cognitive function recovery in the chronic stage.

### Participants

A total of 64 participants including 25 males (age: 46 ± 14.6 years) and 39 females (age: 45 ± 15.7 years) who were experiencing chronic symptoms due to a mild TBI (chronic mTBI) and 40 matched CN comprising 21 males (age: 41 ± 9.4 years) and 19 females (age: 39 ± 10.6 years) participated in this study. mTBI itself was defined by the Mayo Classification System for Traumatic Brain Injury Severity, in which an injury was classified as mild if it met the following criteria: loss of consciousness <30 min, amnesia for <24 h, and no abnormal MRI findings (Malec et al., 2007). Participants had to report a history of one or more prior TBIs (one or multiple) meeting these criteria for mild TBI and have no structural injury to the brain such as a hematoma, contusion, dura penetration, or brain stem injury. They had to meet ICD-10 criteria for chronic mTBI (i.e., post-concussion syndrome) based upon symptoms that were the result of the TBI and could include headache, dizziness, irritability, cognitive problems, emotional problems (e.g., depression or anxiety), hypersensitivity to auditory or visual stimuli, balance problems, insomnia, or other subjective complaints specifically associated with the TBI. Patients also had to report chronic symptoms defined as lasting for at least 6 months from the most recent TBI.

Written informed consent, approved by the Thomas Jefferson University Institutional Review Board, was obtained from all participants, and the study was registered on clinicaltrials.gov with the following identifier: NCT03241732. Participants were recruited from the local community by self-referral and from local neurology offices and were excluded if they had a history of other neurological disorders, significant medical illness, a current substance-use disorder, or current Diagnostic and Statistical Manual of Mental Disorders, 4th Edition (DSM-IV) Axis I psychiatric illness.

For the CN group, individuals were excluded if they had a history of previous TBI, a history of other neurological disorders, significant systemic medical illness, a current substance-use disorder, and current Diagnostic and Statistical Manual of Mental Disorders, 4th Edition (DSM-IV) Axis I psychiatric illness.

### Neuropsychological Assessment

Clinical assessment of TBI participants experiencing chronic symptoms included a battery of self-reported measures including the State-Trait Anxiety Inventory, Beck Depression Inventory, Profile of Mood Scale, Rivermead Post-Concussion Symptoms Questionnaire (RPQ-3 and RPQ- 13), the Epworth Sleepiness Scale, and two cognitive tests—the forward and backward digit span, and the trail making A and B test (Muller et al., 2021). Clinical assessments were performed on the same day as the imaging study. The correlation of neuropsychological tests with the rs-fMRI metrics was investigated across the gray matter area.

### Imaging Protocol

Resting-state fMRI data were obtained from all the participants using a 3T Siemens Biograph mMR Positron Emission Tomography-MR (mMR PET-MR) scanner with a 32-channel head coil. An anatomical SAG T1 MPRAGE was obtained for all the participants to check whether or not any conventional positive radiological findings of brain injury could be detected and to enable further segmentation and registration steps during data preprocessing. MRI parameters for the anatomical T1-weighted sequence were as follows: repetition time = 1.6 s, echo time = 2.46 ms, field of view = 250 mm × 250 mm, matrix = 512 × 512, voxel size = 0.49 × 0.49, 176 slices with slice thickness = 1 mm.

Next, a resting-state BOLD scan was administered using an echo planar imaging (EPI) sequence to examine the intrinsic FC of the brain regions. The following imaging parameters were used: FOV = 23.6 cm; voxel size = 3 mm^3^ × 3 mm^3^ × 4 mm^3^; TR = 2.0s; TE = 30 ms; slice thickness = 4 mm; number of slices = 34; number of volumes = 180; and acquisition time = 366 s. During rs-fMRI, the participants were instructed to close their eyes, keep their heads still, and rest quietly without thinking about anything.

### Data Processing

All rs-fMRI data were preprocessed using Data Processing & Analysis for Resting-State Brain Imaging (DPABI, V5.1_201201)^1^ based on Statistical Parametric Mapping (SPM12)^2^ running on MATLAB R2020b (The Math Works, Inc., Natick, MA, United States) (Yan et al., 2016). The preprocessing steps are listed as follows: the first 10 volumes were discarded to allow magnetization to reach steady state and acclimatization of participants in the scanning environment. The remaining volumes were corrected for slice timing and head motion using six rigid body motion parameters. Next, for each individual, T1-weighted structural image was co-registered to the mean of the realigned EPI images. All T1 images were evaluated by a neuroradiologist to ensure that there were no obvious structural parenchymal abnormalities that would lead to exclusion of the subject and scan from the study and analysis. Then, all resting-state data were spatially normalized to the EPI template in Montreal Neurological Institute (MNI) space with a resampling voxel size of 3 mm × 3 mm × 3 mm. Due to the sensitivity of rs-fMRI measurements to micro head motions, the Friston 24-parameter model (the 24 parameters including 6 head motion parameters, 6 head motion parameters one time point before, and the 12 corresponding squared items) was applied to regress out the head motion effects from the realigned data (Friston et al., 1996). Further, signal from white matter and cerebrospinal fluid were regressed out and filtered with a temporal band-pass of 0.01–0.08 Hz to reduce the effects of low-frequency drifts and high-frequency respiratory and cardiac noise.

### Fractional Amplitude of Low-Frequency Fluctuation Calculation

For each participant, before fALFF calculation, spatial smoothing [Gaussian kernel of full-width half maximum (FWHM) = 6 mm] was performed. Then, with the FFT, the time courses of rs-fMRI signal were converted to frequency domain, and the square root of the power spectrum was measured and averaged across the 0.01–0.08 Hz domain. Then, voxel-wise fALFF was measured as the ratio of power in low-frequency band (0.01–0.08 Hz) to the power of the entire frequency range (0–0.55 Hz). The idea of ALFF was initiated due to the intensity of regional spontaneous brain activity at rest. As such, fALFF introduced measurement showing higher sensitivity and specificity as well as less inclusion of artifacts from vascular signals compared with ALFF (Zou et al., 2008). To ensure standardization, for each participant, the fALFF of each voxel was divided by the global mean fALFF, and standardized fALFF maps were obtained.

### Regional Homogeneity Calculation

Regional homogeneity measurement was performed after filtering by band-pass (0.01–0.08 Hz). This is accomplished on a voxel-based basis by calculating KCC for a given time series that is assigned as the center voxel with those of its nearest 27 neighboring voxels. The equation for calculating the KCC value has been demonstrated in a previous study (Zang et al., 2004). For standardization purpose, ReHo value at each voxel was divided by the global mean of ReHo, and standardized ReHo maps were generated. Then, spatial smoothing with an isotropic Gaussian kernel of 6 mm FWHM was performed after ReHo calculation.

### Functional Connectivity Analysis

Seed-based voxel-wise FC analysis was carried out after band-pass filtering of 0.01–0.08 Hz. The linear trend was removed with the aim of weakening the linear drift of rs-fMRI signal time series. Based on fALFF and ReHo results, the regions that showed a significant difference between mTBI and CN groups were defined as the seeds. The seed regions were created as a spherical 5-mm region of interest (ROI) around the center of mass coordinates where fALFF and ReHo showed significant differences. Then, FC was measured using Pearson’s correlation coefficient between time series of the seeds and the rest of the time series in the gray matter area. For standardization purpose, a Fisher’s *z*-transform was applied to change FC to *z* values, and standardized FC maps were obtained. The converted *z*-score FC maps were referred to as the Pearson correlation coefficient maps.

### Statistical Analysis

To obtain fALFF, ReHo, and seed-based FC differences between the two groups, voxel-based two-sample *t*-tests were performed using the statistical analysis module in the DPABI-V5.1 toolkit. Sex and age were used as covariates in all the two-sample *t*-tests. The resulting statistical maps were corrected for multiple comparisons to a significant level of *p* < 0.001 using false discovery rate (FDR) correction. A chi-square test was applied to estimate the distribution of gender composition in each group. A two-sample *t*-test was conducted to evaluate the effect of age between two groups (*p* < 0.05). Additionally, a one-sample *t*-test was employed on clinical scores among the patient group. Partial correlation analysis was conducted to estimate the correlation between the neuropsychological scores and the rs-fMRI metrics, controlling for sex and age as the covariates. The clusters showing significant group differences were selected as ROIs. The average of fALFF, ReHo, and FC was extracted over the mask of ROIs. Spearman’s rank correlation coefficients between the mean values of fALFF, ReHo, and seed-based FC and neurophysiological scores were generated for all the patients within the TBI cohort (*n* = 64). A significance value was determined using a student’s *t* distribution, with the linear correlation being considered significant if *p*-value was less than 0.05. In all steps of the data processing, the name of the significant brain regions was recorded according to the Automated Anatomical Labeling (AAL) atlas (Tzourio-Mazoyer et al., 2002).

## Results

### Demographic and Clinical Characteristics

The demographic and clinical characteristics of all the participants are shown in Table 1. Significant difference between the age of CN and mTBI groups was observed (two-sample *t*-test, *p*-value = 0.03). However, no evidence of a difference in the proportion of males and females was found in each group (CN: chi-square, χ^2^ = 0.1, *p*-value = 0.75; mTBI: chi-square, χ^2^ = 3.6, *p*-value = 0.08). Additionally, we found a significant difference for all the clinical scores within the patient group (one-sample *t*-test, *p*-value < 0.001).

**TABLE 1:** Participant demographic and neuropsychological measures, averages, and standard deviations reported among mTBI and CN groups.

	CN	mTBI	*p*-value	Statistics
**Demographics**	*n* = 40	*n* = 64		
Age (year) (SD)	40.3 (9.9)	46.0 (14.8)	0.03^a^	*T* = −2.1^a^
Sex (M/F)	21: 19	25: 39		
			CN: 0.75^b^	χ^2^ (CN): 0.1^b^
			mTBI: 0.08^b^	χ^2^ (mTBI): 3.6^b^
Injury-to-imaging interval (months)(std)	–	75.0 (72.8)		
Single concussion vs. multiple (single: multiple)	–	19: 45		

**Neuropsychological measures**	**(Mean ± SD)**	***p*-value**	**Statistics**

**State Trait Anxiety Inventory**			
State Anxiety	46.1 ± 13.4	<0.001^c^	T = 19.3^c^
Trait Anxiety	46.4 ± 12.0	<0.001^c^	T = 21.7^c^
Back Depression Inventory	17.1 ± 11.0	<0.001^c^	T = 9.2^c^
**Profile of Moods Scale**			
Tension	12.6 ± 8.4	<0.001^c^	T = 8.4^c^
Depression	13.8 ± 15.2	<0.001^c^	T = 5.1^c^
Anger	10.2 ± 8.8	<0.001^c^	T = 6.5^c^
Vigor	9.8 ± 6.1	<0.001^c^	T = 9.8^c^
Fatigue	12.2 ± 7.2	<0.001^c^	T = 9.0^c^
Confusion	11.4 ± 5.9	<0.001^c^	T = 10.9^c^
**Mayo-Portland Adaptability Inventory-4**			
Ability Index	14.9 ± 9.4	<0.001^c^	T = 8.9^c^
Adjustment Index	17.9 ± 9.1	<0.001^c^	T = 11.1^c^
Participation Index	9.4 ± 6.3	<0.001^c^	T = 8.4^c^
Total	35.8 ± 19.7	<0.001^c^	T = 10.2^c^
**Rivermead**			
RPQ-3	5.6 ± 2.8	<0.001^c^	T = 15.3^c^
RPQ-13	28.2 ± 9.6	<0.001^c^	T = 23.3^c^
Digit Span			
Forward	10.7 ± 2.3	<0.001^c^	T = 37.2^c^
Backward	7.3 ± 2.5	<0.001^c^	T = 23.4^c^
Epworth Sleepiness Scale	7.9 ± 5.1	<0.001^c^	T = 12.4^c^
**Trail Making (seconds to complete)**			
A	30.1 ± 14.6	<0.001^c^	16.3^c^
B	65.0 ± 27.7	<0.001^c^	18.6^c^

*CN, healthy control; mTBI, mild traumatic brain injury; RPQ, Rivermead Post-Concussion Symptoms Questionnaire; SD, standard deviation.*

*^a^p-value and T-statistic obtained by two-sample t-test.*

*^b^p-value and χ^2^-statistic obtained using chi-square t-test.*

*^c^p-value and T-statistic obtained by one-sample t-test.*

### Fractional Amplitude of Low-Frequency Fluctuation Differences Among the Groups

**FIGURE 1:**
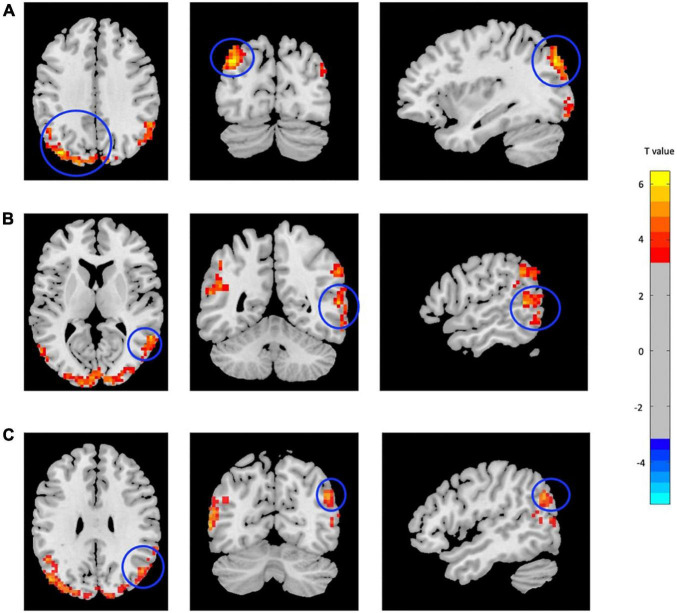
Increased fALFF in mTBI patients compared with controls in the clusters including **(A)** Occipital_Mid_L, **(B)** Temporal_Mid_R, and **(C)** Angular_R (*p*-value <0.001, FDR-corrected). The range of *t*-values is represented by the color bar. Hot colors corresponding with increased fALFF values in the mTBI group. *Note that the right side of the image as displayed is the right side of the person.

Compared with CN, mTBI patients showed increased fALFF in three main clusters including left middle occipital cortex (MOC), right middle temporal cortex (MTC), and right angular gyrus (AG) (*p*-value < 0.001, FDR-corrected). The list of the significant clusters identified by the two-sample *t*-test is shown in Figure 1 and Table 2.

**TABLE 2:** Regions with significant fALFF differences between mTBI patients and CN.

Brain region (AAL)	Cluster size (voxels)	Peak MNI coordinate (x, y, z)	*T* value
Occipital_Mid_L	1,226	−36, −78, 36	6.48
Temporal_Mid_R	115	57, −51, 9	5.72
Angular_R	115	48, −66, 30	5.51

*fALFF, fractional amplitude of low-frequency fluctuations; mTBI, mild traumatic brain injury; CN, healthy control; ALL, Automated Anatomical Labeling; MNI, Montreal Neurological Institute.*

*T, statistical value of peak voxel.*

*x, y, z, coordinates of primary peak locations in the space of MNI.*

*L, left; R, right.*

*Comparison was performed at voxel-level p < 0.001, FDR-corrected.*

*The voxel T threshold for voxel p threshold 0.001 was 3.17.*

### Regional Homogeneity Differences Among the Groups

**FIGURE 2:**
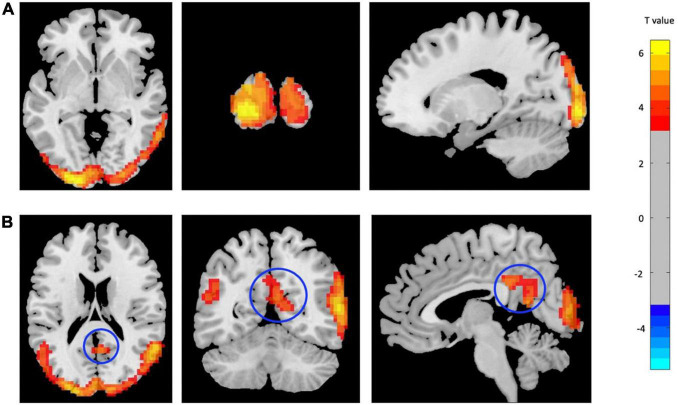
Increased ReHo in mTBI patients compared with controls in the clusters including **(A)** Occipital_Mid_L, and **(B)** Cingulate_Post_L (*p*-value <0.001, FDR-corrected). The range of *t*-values is represented by the color bar. Hot colors corresponding with increased ReHo values in the mTBI group. *Note that the right side of the image as displayed is the right side of the person.

Two main significant clusters showing the difference between mTBI patients compared with CN are shown in Figure 2 and Table 3. The two-sample *t*-test demonstrated significantly increased ReHo in the clusters located in the left MOC and left posterior cingulate gyrus cortex (PCC) in mTBI patients compared with CN (*p*-value < 0.001, FDR-corrected).

**TABLE 3:** Regions with significant ReHo differences between mTBI patients and CN.

Brain region (AAL)	Cluster size (voxels)	Peak MNI coordinate (x, y, z)	*T* value
Occipital_Mid_L	2,852	−18, −96, −3	6.71
Cingulate_Post_L	247	0, −36, 30	6.18

*ReHo, regional homogeneity; mTBI, mild traumatic brain injury; ALL, Automated Anatomical Labeling; MNI, Montreal Neurological Institute; CN, healthy control. T, statistical value of peak voxel.*

*x, y, z, coordinates of primary peak locations in the space of MNI.*

*L, left; R, right.*

*Comparison was performed at voxel-level p < 0.001, FDR-corrected.*

*The voxel T threshold for voxel p threshold 0.001 was 3.2.*

### Seed-Based Functional Connectivity Differences Among the Groups

**FIGURE 3:**
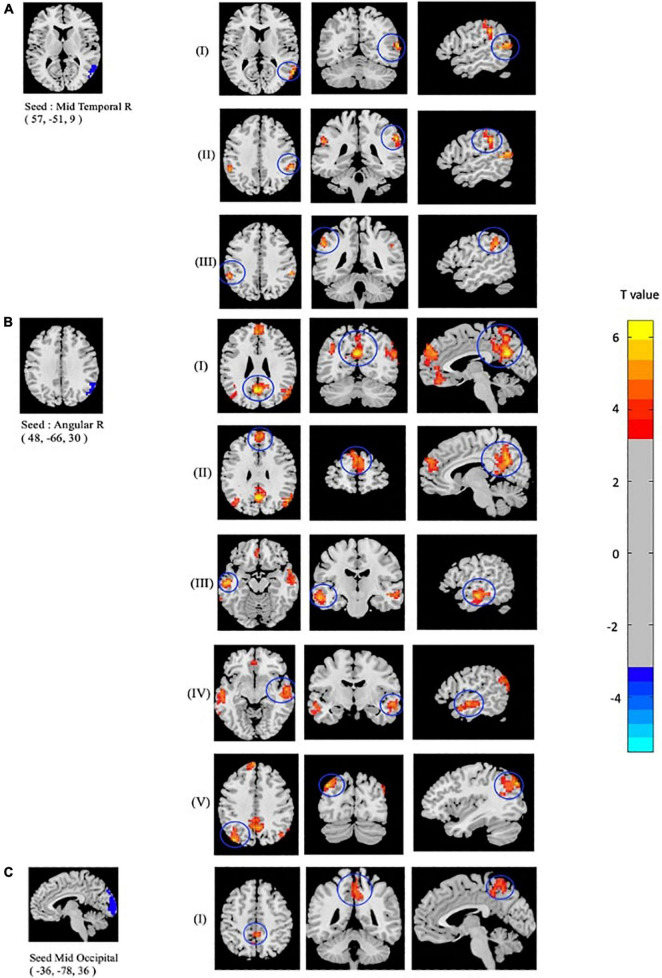
Altered seed-based functional connectivity (FC) in mTBI patients compared with controls including increased FC between the seed located at **(A)** Temporal_Mid_R, and clusters located at (I): Temporal_Sup_R, (II): SupraMarginal_R, and (III): Parietal_Inf_L, as well as the seed located at **(B)** Angular_R and clusters located at (I): Precuneus_L, (II): Frontal_Sup_Medial_L, (III): Temporal_Mid_L, (IV): Temporal_Sup_L, (V): and Occipital_Mid_L. In addition, altered FC between the seed located at **(C)** Occipital_Mid_R and (I): Precuneus_R is shown (*p*-value <0.001, FDR-corrected). The range of *t*-values is represented by the color bar. Hot colors corresponding with increased seed-based FC values in the mTBI group. *Note that the right side of the image as displayed is the right side of the person.

As mentioned above, the center points of the peak *t*-value where brain regions showed significant differences in fALFF and ReHo values between mTBI patients and CN were defined as spherical seeds (*r* = 5 mm) including the left MOC, right MTC, right AG, and left PCC. Further, seed-based FC was estimated between these four seeds and the rest of the brain in gray matter areas. Compared with CN, increased FC was identified between the seed located at the left MOC and right precuneus in mTBI. When the right MTC was used as the seed, an increased FC pattern was observed between the MTC and right superior temporal cortex (STC), right supramarginal gyrus, and left inferior parietal cortex (IPC) in mTBI patients. Moreover, an increased FC pattern was found between the seed located at the right AG and left superior medial frontal cortex (SMFC), left MTC, left superior temporal cortex (STC), right AG, left MOC, and left precuneus in the patient cohort (*p*-value < 0.001, FDR-corrected). The list of significant seed-based FC group differences measured by the two-sample *t*-test is shown in Figure 3 and Table 4.

**TABLE 4:** Regions with significant seed-based FC differences between mTBI and CN.

Seed	Peak MNI coordinate (seed)	Brain region (AAL)	Cluster size (voxels)	Peak MNI coordinate (x, y, z)	*T* value
Temporal_Mid_R	57, −51, 9	Temporal_Sup_R	99	63, −54, 21	6.39
		SupraMarginal_R	66	57, −36, 36	6.13
		Parietal_Inf_L	58	−54, −42, 39	5.83
Angular_R	48, −66, 30	Precuneus_L	621	0, −60, 27	6.73
		Frontal_Sup_Medial_L	361	0, 51, 30	5.90
		Temporal_Mid_L	210	−60, −18, −15	6.23
		Temporal_Sup_R	208	57, −9, −9	5.70
		Occipital_Mid_L	198	−39, −78, 39	6.35
Occipital_Mid_L	−36, −78, 36	Precuneus_R	176	3, −42, 48	5.78

*FC, functional connectivity; mTBI, mild traumatic brain injury; CN, healthy control; ALL, Automated Anatomical Labeling; MNI, Montreal Neurological Institute. T, statistical value of peak voxel.*

*x, y, z, coordinates of primary peak locations in the space of MNI.*

*L, left; R, right.*

*Comparison was performed at voxel-level p < 0.001, FDR-corrected.*

*The voxel T threshold for voxel p threshold 0.001 was 3.2.*

### Correlation Analysis

The clinical status regarding fALFF, ReHo, and seed-based FC values was estimated using Spearman rank correlations among the patient cohort. The regional fALFF and ReHo were extracted from the left MOC, right MTC, right AG, and left PCC. Then, partial correlations were estimated between neuropsychological performance and the rs-fMRI metrics, controlling for age and gender. The result of the correlation analysis showed that fALFF (*r* = −0.43, *p* = 0.001) and ReHo (*r* = −0.34, *p* = 0.008) values in the left MOC were negatively correlated with trail making A. Also, fALFF in the cluster of the right AG showed negative correlation with RPQ-13 (*r* = −0.26, *p* = 0.045) and trail making A scores (*r* = −0.39, *p* = 0.002). fALFF (*r* = −0.28, *p* = 0.029) and ReHo (*r* = −0.32, *p* = 0.012) values in the right MTC showed a negative correlation with trail making A scores. ReHo values extracted from the left PCC were negatively correlated with state anxiety (*r* = −0.41, *p* = 0.022), trail anxiety (*r* = −0.45, *p* = 0.012), tension (*r* = −0.39, *p* = 0.032), depression (*r* = −0.46, *p* = 0.011), fatigue (*r* = −0.407, *p* = 0.026), confusion (*r* = −0.41, *p* = 0.024), and trail making A scores (*r* = −0.37, *p* = 0.004).

**FIGURE 4:**
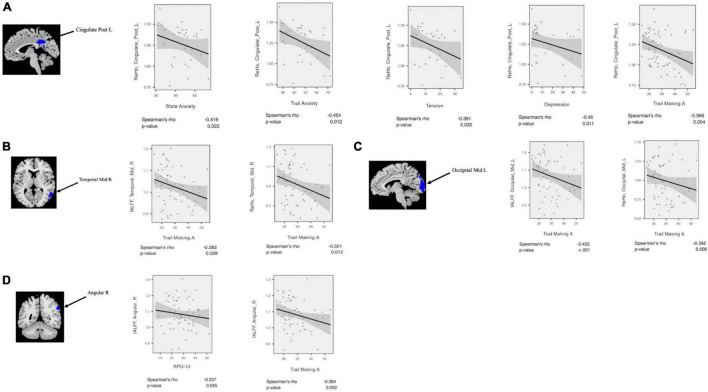
Correlation analysis between fALFF/ReHo and neuropsychological scores among mTBI patients in the clusters with significant fALFF/ReHo differences between mTBI patients and controls including **(A)** Cingulate_Post_L, **(B)** Temporal_Mid_R, **(C)** Occipital_Mid_L, and **(D)** Angular_R.

**FIGURE 5:**
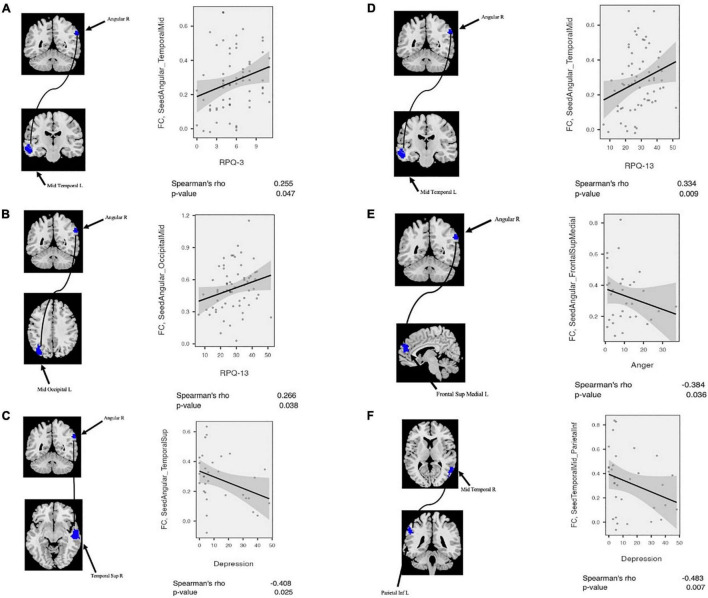
Correlation analysis between altered seed-based functional connectivity (FC) and neuropsychological scores among mTBI patients. **(A)** RPQ-3 is positively correlated with altered connectivity between Angular_R and Temporal_Mid_L. **(B)** RPQ-13 is positively correlated with altered connectivity between Angular_R and Temporal_Mid_R. **(C)** Depression score from POMS is negatively correlated with altered connectivity between Angular-R and Temporal_Sup_R. **(D)** RPQ-13 is positively correlated with altered connectivity between Angular_R and Temporal_Mid_R. **(E)** Anger score from POMS is negatively correlated with altered connectivity between Angular_R and Frontal_Sup_Medial_L. **(F)** Depression score from POMS is negatively correlated with altered connectivity between Temporal_Mid_R and Parietal_Inf_L. POMS, profile of mood states.

Likewise, correlation analysis between seed-based FC and neuropsychological scores was performed. As mentioned above, regarding FC correlation analysis, the ROIs were defined in the clusters of significant differences between patient and CN (*p*-value < 0.05, FDR-corrected). Then, the average of the seed-based FC was extracted from the ROIs. Within the mTBI cohort, correlation analysis showed a positive association between RPQ-3 and altered FC between the right AG and left MTC (*r* = 0.26, *p* = 0.047), a positive association between RPQ-13 and altered FC between the right AG and left MOC (*r* = 0.27, *p* = 0.038), a positive association between RPQ-13 and altered FC between the right AG and right MTC (*r* = 0.34, *p* = 0.009), a negative correlation between anger and altered FC between the right AG and left SMFG (*r* = −0.39, *p* = 0.036), a negative correlation between depression and altered FC between the right AG and right STC (*r* = −0.41, *p* = 0.025) and altered FC between the right MTC and left IPC (*r* = −0.49, *p* = 0.007). The results of the correlation analysis between the rs-fMRI metrics and seed-based FC with the neuropsychological scores are shown in Figures 4, 5.

## Discussion

In this study, we investigated the association between neuropsychological performance in chronic mTBI patients and altered neural activity at two levels, with fALFF and ReHo at the local level and FC at the network level. To date, relatively few studies have examined the cognitive function in patients with mTBI and its correlation with rs-fMRI metrics. Compared to controls, our findings demonstrated increased fALFF in the left MOC, right MTC, and right AG in the chronic mTBI patients. Moreover, we have found increased ReHo in the patient group located in the left MOC and left PCC.

The temporal and occipital cortices have been known to be at risk of contusion in moderate to severe TBI. Our study showed higher fALFF in the right MTC and left MOC. Likewise, several studies reported elevated ALFF/fALFF activity in several temporal and occipital regions at rest in patients with mTBI and mild cognitive impairment (Qi et al., 2010; Han et al., 2011; Zhan et al., 2016). It has been shown that fALFF is typically higher in cortical rather than in relative subcortical regions. fALFF is also associated with increased metabolism in cortical regions. Higher fALFF and metabolism in cortical areas could be interpreted as representing a potential compensatory response to the damage in patients suffering from trauma (Stephenson et al., 2020). In line with this, a recent study in patients with severe TBI showed higher ALFF in the right temporal, frontal, and IPC in the conscious subgroup compared with the coma subgroup. They concluded that increased ALFF values in these regions are associated with strong spontaneous activities which may compensate for severe structural damages and reconstruction of FC among these areas (Guo et al., 2019). Additionally, a previous study found increased spontaneous neural activity in the occipital region in mTBI patients compared with CN. These authors proposed that the occipital region as a part of the visual brain network is active at rest which could be associated with processing visual imagery unconsciously and experiencing mental images of the trauma during resting-state scanning (Zhan et al., 2016). It has been shown that neural activity in the temporal region is associated with semantic processing including memory, visual, verbal, and executive functioning (Konstantinou et al., 2018). A previous study reported decreased ALFF in the temporal cortex at resting state in patients with TBI compared with CN (Zhou et al., 2014). Another study showed that ALFF/fALFF measures could be correlated with better cognitive performance in patients with chronic mTBI (Palacios et al., 2013). Zhan et al. (2016) reported that reduced spontaneous neural activity in acute mTBI patients might be linked with emotional and cognitive disorders (Zhan et al., 2016). fALFF and ReHo are common analytical methods used for investigating the pathophysiology of various neuropsychological disorders (Zhan et al., 2015; Yang et al., 2018; Fu et al., 2019; Wang et al., 2019; Che et al., 2020; Mo et al., 2020). In this study, we took advantage of these tools to explore the neurological mechanism of chronic mTBI and its relationship with cognitive performance. Altered rs-fMRI metrics in the default mode network (DMN) after mTBI have been consistently reported in the literature (Cole et al., 2010; Meier et al., 2017). For instance, a previous study reported increased ReHo in the anterior portion of the DMN following mTBI in adults with sport-related concussion (Meier et al., 2020). However, another study found decreased ReHo located among frontal, temporal, and parietal lobes and limbic regions including the left insula that was significantly correlated with cognitive damage. The authors suggested that decreased ReHo in acute mTBI patients could be associated with reduced ability to do executive functions (Zhan et al., 2015).

The AG is one of the main components of the DMN and a part of the parietal cortex. This is linked with cognition, memories, and visual word forms (Price, 2000). In addition, this supports the automatic mapping between mathematical symbols and semantic referents. The anterior temporal lobe and AG are the components of a semantic processing system. As such, alteration of spontaneous neural activity in these regions could be linked with cognitive dysfunction (Price, 2000; Grabner et al., 2013).

A recent study in a group of patients with posttraumatic stress disorder (PTSD) showed decreased ReHo values in the right AG compared with controls. They also have found a negative correlation between decreased ReHo values in the right AG and symptom severity of PTSD (CAPS). The researchers concluded that ReHo alteration in the AG is a common feature of PTSD that could be associated with dissociative symptoms of PTSD (Fu et al., 2019). The results of our study are consistent with this conclusion, as we have observed increased ReHo values in the right AG that were negatively associated with clinical scores including RPQ-13 and trail making A. However, the role of the AG in TBI and PTSD disorders is not yet clearly understood, but we speculate that the alteration of structural and functional connections of the AG may be engaged in neurobiological change in traumatic disorders.

The posterior components of the DMN, particularly PCC, are vulnerable to injury in patients with TBI (Zhou et al., 2012; Ke et al., 2018). Prior studies reported structural and FC alteration in the PCC in patients suffering from mTBI (Yount et al., 2002; Stillman et al., 2008). It has been shown that the PCC is activated during spontaneous thoughts and across a variety of social, memory, and emotional tasks (Andrews-Hanna, 2012). Consistent with these findings, we found that increased ReHo in the PCC is negatively correlated with chronic cognitive symptoms of mTBI, including state anxiety, trait anxiety, trail making A, tension, depression, fatigue, and confusion scores.

Emotional disruption including anxiety, fatigue, depression, and apathy has been known as a common non-motor symptom in patients suffering from TBI. Previous literature has confirmed the disturbance of the emotional processing circuits in chronic mTBI patients (Sharp et al., 2011). Our study translated these findings into a real-world clinical setting and proposed that higher local neural connectivity in the PCC was linked with cognitive and emotional recovery mechanism in the patient group.

We proposed that the higher fALFF and ReHo values in the chronic mTBI group may be caused by greater neural activity and local neuronal FC that is associated with transmitting information to other brain areas and increased local connectivity among the regions. This phenomenon could be due to neuroplasticity and compensatory mechanism in response to the injury that is linked with worse cognitive performance, reflecting both impairment and maladaptation through the progression of the disease.

From the FC aspect, we found increased seed-based FC between the seed located at the right MTC, right STC, right supramarginal, and left IPC. Altered FC between the right MTC and the left IPC was also negatively correlated with depression scores. Previous studies have shown that mTBI is associated with brain FC alteration through the DMN, motor, salient, central executive, and visual networks. Indeed, the DMN is responsible for the retrospective state of mind at rest in which people are alert and conscious. Hence, decoupling of FC between anterior and posterior of the DMN nodes could be interpreted as the loss of conscious awareness at rest (Mak et al., 2017; Tagliazucchi and van Someren, 2017). A previous study showed increased functional and structural connectivity between subnetwork nodes in the DMN including the temporal pole, superior temporal gyrus, temporal lobe, and PCC in patients with chronic mTBI 1 year after injury (Dall’Acqua et al., 2017). Another study reported increased FC between the prefrontal and posterior parietal cortex in a group of patients with chronic mTBI. Furthermore, the authors proposed that hyperconnectivity in the mTBI group at rest might be associated with increased awareness of the external environment and excessive cognitive fatigue (Shumskaya et al., 2012).

From the seed-based FC analysis, we also found increased FC between the seed located at the right AG and the left precuneus, left MSFC, left MTC, left STC, and left MOC in the patients compared with controls. In addition to the FC alteration in the patient group, we found a positive correlation between the AG-MTC connectivity and RPQ-3 and RPQ-13 scores, as well as a positive correlation between AG-MOC connectivity and RPQ-13 scores, suggesting that the uncoupling between the AG-MTC and AG-MOC might be associated with the disrupted cognitive self-regulation in chronic mTBI. Additionally, we observed a significant negative correlation between depression and altered FC between the right AG and right STC, as well as a negative correlation between anger and altered FC between the right AG and left MSFC, suggesting that hyperconnectivity after brain injury may be linked with cognitive recovery and efficient behavioral responses.

Our findings agree well with previous literature suggesting enhanced FC between the AG and prefrontal cortex in acute mTBI patients (Marquez de la Plata et al., 2011). In addition, hyperactivity within DMN nodes has been reported in patients with acute mTBI and has been found to be related to working memory dysfunction and attention switching during cognitive demand (Bernier et al., 2017; Li et al., 2020). Sharp et al. (2011) also examined resting-state FC in the DMN in patients with mTBI and found greater connectivity between posterior cingulate and precuneus with the rest of the DMN in patients when compared with CN (Sharp et al., 2011). They proposed that greater FC between posterior cingulate and the rest of the DMN is linked with an adaptive response to cognitive impairment. In line with this, our results also showed increased FC between the seeds located in the right MOC and the right precuneus. Taken together, our results demonstrate that high resting-state FC after traumatic brain injury may be linked with less disruption in information processing through cognitive recovery. Additionally, the results suggest that sustained changes in FC, particularly in the DMN nodes, might affect behavior by modulating the responsiveness of the brain networks through cognitive task performance.

Several limitations also need to be considered in the interpretation of our study results. First, the discrepancies in age and sex of controls and patient groups may be a confounding factor in the between-group differences. Even though we have included sex and age of the participants as covariates in within-group and between-group analyses, selecting patients from a homogeneous group could ensure more robust outcomes. Indeed, heterogenous factors causing injuries such as accidents, falls, and blast could lead to different types of mTBI. Furthermore, the patients had a diverse duration between their most recent mTBI and participation in the study. Hence, it is likely that different types of injuries lead to various neurobiological consequences as well as divergent compensation and recovery mechanisms. Second, this study compared a small sample size and unequal numbers of CN and mTBI cohorts. Future experiments are recommended to contain larger sample sizes and an equal number of participants for both groups of controls and patients. Additionally, clinical scores were acquired just for the patient group in this study. However, future studies might take this into account to obtain clinical scores from both healthy control and patient groups and examine the association of rs-fMRI measurements difference between the groups with respect to changes in the scores. This would potentially provide a better indication of functional compensation or restoration among the patient group. Finally, while our results suggest the association of better cognitive performance with increased FC, it is still not exactly clear how the direction of this relationship works. More homogenous experiments are needed to investigate the association between rs-fMRI metrics and cognitive performance using longitudinal studies at different time points after injury.

## Conclusion

Our study highlights the utility of data-driven resting-state analysis among patients suffering from chronic mTBI. The results suggest that chronic mTBI patients have altered BOLD signal using fALFF and ReHo and FC parameters as the robust and sensitive clinical biomarkers. Moreover, we provided evidence of cognitive performance associated with spontaneous brain activity at rest, particularly in the DMN, visual network, and emotional processing circuits, at the chronic stage after the brain injury. Generally, our results emphasize the importance of exploring DMN and cognitive circuits following brain injury. We propose that the recovery from the brain injury captured by rs-fMRI measurements can be linked with clinical recovery according to the standard clinical cognitive assessments.

## Data Availability Statement

The raw data supporting the conclusions of this article will be made available by the authors, without undue reservation.

## Ethics Statement

The studies involving human participants were reviewed and approved by Thomas Jefferson University Institutional Review Board. The patients/participants provided their written informed consent to participate in this study.

## Author Contributions

FV: conceptualization, methodology, validation, formal analysis, investigation, resources, data curation, writing—review and editing, visualization, supervision, and project administration. AN: conceptualization, methodology, validation, resources, project administration, funding acquisition, investigation, resources, and writing—review and editing. MA: methodology, validation, formal analysis, investigation, data curation, and writing—review and editing. JM, SS, and DMi: methodology, validation, and formal analysis. GZ, NW, and AJB: resources, project administration, and funding acquisition. DMo: conceptualization, resources, project administration, and funding acquisition. FM: conceptualization, methodology, data curation, writing—review and editing, supervision, and project administration. All authors read and approved the final manuscript.

## Conflict of Interest

The authors declare that the research was conducted in the absence of any commercial or financial relationships that could be construed as a potential conflict of interest.

## Publisher’s Note

All claims expressed in this article are solely those of the authors and do not necessarily represent those of their affiliated organizations, or those of the publisher, the editors and the reviewers. Any product that may be evaluated in this article, or claim that may be made by its manufacturer, is not guaranteed or endorsed by the publisher.

## Footnotes

1
http://rfmri.org/dpabi

2
http://www.fil.ion.ucl.ac.uk/spm
